# Human papillomavirus detection in head and neck squamous cell carcinoma

**DOI:** 10.3332/ecancer.2014.475

**Published:** 2014-10-23

**Authors:** Dayahindara Vietía, Juan Liuzzi, Maira Ávila, Zoraya De Guglielmo, Yrneh Prado, María Correnti

**Affiliations:** 1 Laboratorio de Genética Molecular, Instituto de Oncología y Hematología-MPPS, Caracas 1050, Venezuela; 2 Servicio de cabeza y cuello del Hospital Oncológico ‘Padre Machado’-IVSS, Caracas 1050, Venezuela

**Keywords:** HPV, squamous, carcinoma, head, neck

## Abstract

**Introduction:**

Human Papillomavirus (HPV) has been associated with benign and malignant lesions in different epitheliums. The relationship between specific genotypes of high-risk HPV and some human cancers is well established. The aim of this work was to detect the HPV genotypes present in head and neck squamous cell carcinoma (HNSCC).

**Methods:**

We evaluated 71 samples of patients with histopathological diagnosis of HNSCC. The DNA extraction was conducted with the QIAGEN commercial kit. HPV detection and genotyping were performed by reverse hybridisation (INNO-LiPA) following the commercial specifications.

**Results:**

The mean age of the patients evaluated was 60.7 ± 13.11 years. The distribution of the lesions included 25 (35.20%) cases of squamous cell carcinoma (SCC) of the oral cavity, 23 (32.39%) of larynx, 16 (22.50%) of the oropharynx, 4 (5.63%) of paranasal sinus, and 2 (2. 80%) cases of SCC of the nostril. Of the patients, 78.9% were males, and of these 76% were tobacco users and 67.6% were alcohol consumers. The viral DNA was detected in 67.6% of the samples. The oral cavity and the larynx were the highest HPV-positivity sites with 35.40% and 29.10% respectively. The most frequent genotype was 16 as single infection (18.70%), or in combination with another HPV types. In the oral cavity and larynx the genotypes 16 or the combination 6 and 51 were present in 11.76% and 14.28%, respectively; and in the oropharynx the most frequent genotype was 16 in 22.50% of the cases, and in the paranasal sinus 50% presented infection with HPV-6. We observed that tumours with most advanced size and stage presented greater HPV positivity.

**Conclusions:**

This study shows a high percentage of HPV positivity in SCC is mainly associated with high-risk HPV. It is important to highlight that viral infection, especially HPV-16, could be a risk factor in HNSCC progression.

## Introduction

Head and neck squamous cell carcinoma (HNSCC) is the eighth most common malignancy in the world, with approximately 620,000 patients diagnosed each year with cancer of the oral cavity, nasopharynx, oropharynx, and larynx [1]. The disease is characterised by local tumour aggressiveness, early recurrence, and high frequency of second primary tumours [2]. In Venezuela, HNSCC represents 2% of the overall number of malignant tumours, according to data from the Ministerio del Poder Popular para la Salud (MPPS). Apart from the high mortality rate for HNSCC, quality of life is often very problematic for patients with advanced oncological disease in the head and neck area [3]. It is generally accepted that oral carcinogenesis is a multistep process of accumulated genetic damage leading to cell deregulation, with disruption in cell signaling, deoxyribonucleic acid (DNA) repair, and cell cycle; all of which are fundamental for homeostasis [4]. The epidemiology of head and neck cancer has been well described, and multiple risk factors have been identified; however, the molecular mechanisms responsible for the development of these neoplasms remain poorly understood. The first and most important cause of HNSCC development is tobacco consumption. It is believed that 85–90% of these cases had a tobacco and/or alcohol history, and the risk is proportional to consumption [4]. At present, an increment in the incidence of tobacco and alcohol frequency has been suggested, mainly in young adults and women [5]. However, a percentage of these patients are not consumers of alcohol or tobacco, in this group are included young adults and women exposed to other risk factors such as HPV infection and EBV (Epstein-Barr Virus), which have been associated in the past with the head and neck cancer development [6].

Human Papillomavirus (HPV) is a member of the Papillomaviridae family. It has a size of 55 nm and its genome is composed for a circular double DNA strand of about 7.9 kb with a well-preserved general organisation. It is divided into three sections, one section containing the open reading frames (ORF); the other section containing the genes of ‘early’ expression (E1–E7) which highlight E6 and E7 for its oncogenic potential and are expressed shortly after the infection and prior to the onset of DNA; and another section includes the ‘late’ genes (L) involved in the viral capsid formation [7]. In humans, these viruses cause pathologies that range from benign warts (associated with low-risk HPV) to malignant cancer (associated with high-risk HPV). It is now accepted that HPV is the causative agent of more than 90% of all cervical cancers [8]. HPV has also been found to be associated with anal cancer [9], and anogenital warts [10]. In recent years HPV has been associated with cases of HNSCC [11], and in particular high-risk HPV infection may be a risk factor for oropharyngeal cancer [12]. HPV has been suggested to be associated with approximately 25% of HNSCC, having been identified in the oropharynx and particularly in tonsil cancers [13]. Although the mechanism of transmission in the head and neck are not yet clarified, some authors have suggested that these viruses may act as a risk factor in the development of these cancers. Given the consideration that HPV infection is caused primarily by sexual contact, it would not be difficult to arrive at the mouth through oral sex, among other means [14]. In our review we observed that in Venezuela there are very few studies that show the role played by HPV in HNSCC development.

At present the population-level burden of HPV–positive in HNSCC is currently unknown and this could have important implications for cancer prevention, potentially through HPV vaccination. Therefore, in the present study we propose to evaluate HPV infection using the INNO-LiPA HPV Genotyping Extra test in HNSCC patients.

## Materials and methods

### Patients and tumour specimens

Seventy-one patients with a diagnosis of SCC of the oral cavity, larynx, nostril, paranasal sinus, or oropharynx treated at the Head and Neck Service of the Oncologic Hospital ‘Padre Machado’ (IVSS), Caracas-Venezuela were selected. All the patients signed a written informed consent to participate in the study. Fresh biopsies were obtained and cut into two sections, one was frozen at –70 °C for molecular analysis and the other fragment was fixed in 10% neutral formalin and paraffin-embedded. It was processed to obtain 5 µm thick paraffin sections and routinely stained with H&E for histological diagnosis. The assessment of the differentiation grade was made according to Wiernik *et al* (1991) [15]. Clinical information was collected from the medical case history.

### DNA extraction

DNA extraction was performed using the QIAGEN® commercial kit, following the commercial specifications. Biopsies were cut and incubated at 65 °C overnight with ATL buffer, subsequently buffer AL was added and incubated at 72 °C for 10 minutes, and 100% ethanol was added and transferred to a column; washes were performed with buffers W1 and W2. The elution was performed with 200 µL of the elution solution.

### HPV detection

HPV detection was performed using the INNO-LiPA HPV Genotyping Extra kit (Innogenetics), following the commercial specifications. The kit allows specific detection of 25 HPV genotypes (HPV types 6, 11, 16, 18, 31, 33, 35, 39, 40, 42, 43, 44, 45, 51, 52, 53, 54, 56, 58, 59, 66, 68, 70, 73, 74), and is based on reverse hybridisation. The test included a PCR amplification of a 65 bp fragment within the L1 region of the HPV genome using the broad spectrum SPF10 biotinylated primers. Biotinylated amplicons are subsequently hybridised with HPV type-specific oligonucleotide probes which are immobilised as parallel lines on membrane strips. After hybridisation and stringent washing, streptavidin-conjugated alkaline phosphatase is added and bound to any biotinylated hybrid formed. Incubation with BCIP/NBT chromogen yields a purple precipitate, and the results can be visually interpreted.

### Statistical analysis

The HPV genotype specific prevalence was expressed as the portion of HPV-positive carcinoma cases. Categorical variables were studied using chi-squared test. All statistical analyses were performed using SPSS V.2.0.

## Results

The distribution of the 71 lesions included 25 (35.20%) cases of SCC of the oral cavity, 23 (32.39%) of larynx, 16 (22.50%) of the oropharynx, 4 (5.63%) of paranasal sinus, and 2 (2.80%) cases of SCC of the nostril. The mean age of patients was 60.7 years (range 49–83); 78.9% of the patients were males, and of these 76% were tobacco users, and 67.6% were alcohol consumers. Regarding the HNSCC stage; 7.04% were in early stage and 56.33% in advanced stage.

### HPV detection by INNO-LiPA HPV Genotyping Extra test

The HPV types identified by the INNO-LiPA HPV Genotyping Extra test are presented in Table 1. The HPV genome was detected in 48/71 (67.60%) of the HNSCC cases, in these positive cases we identified infection with only one HPV-6 genotype in 18.7% (9/48), 12% (6/48) for HPV-6, two cases for HPV-31, two cases for HPV-35, two for HPV-51 (4.10%) respectively, and one case (2%) was positive for HPV-53. We found multiple HPV-type infections in 26 cases (54.0%); nine samples were positive for HPV-6, HPV-11, and HPV-16, five for HPV-6 and 51, and two were positive for HPV types 6 and 11; two for HPV 6 and 31; which are the most frequent (Table 1).

Regarding the anatomical location of the tumour, the oral cavity and the larynx were the highest HPV-positivity sites, with 35.40% and 29.10% respectively. The most frequent genotypes in both were HPV-16 and HPV-51 (Table 2). 25% of the cases were HPV positive in the oropharynx with 22.50% HPV-16 genotypes, and in the paranasal sinus 8.3% were HPV positive, and of these 50% were HPV-6.

In accordance with tumour development, we could suggest that tumours with the most advanced size and stage presented greater HPV positivity, 42.8% and 57.1% respectively (Table 3).

## Discussion

The incidence of head and neck cancer is strongly linked to alcohol and smoking abuse; however, increasing epidemiologic evidence postulates the existence of a subgroup of these cancers with a different oncogenesis based on HPV infection [6]. HPV-positive oropharyngeal cancers, in particular, may comprise a distinct molecular and pathologic disease entity that is causally associated with HPV infection and has a markedly improved prognosis [16].

The reports of frequency of HPV DNA in HNSCC have varied between 14% and 60%, depending on tumour site, specimens, number of cases included, and methods applied for molecular analysis [15]. The techniques commonly employed for HPV detection is PCR with generic primers MY09/MY11 and GP5+/GP6+, considered as one of the most sensitive; however the length of the amplified region (450pb) is frequently a limitation [17].

The aim of the present study was HPV identification in HNSCC samples, using fresh-frozen specimens in which the DNA might be conserved better than in archived specimens. We demonstrated the presence of HPV in 67.6% cases, employing the INNO-LiPA kit, this agrees with published studies of HNSCCs where the average prevalence of HPV in these patients was 60% and more [18]. Data reported by Koskinen *et al* (2003) evaluated the frequency of HPV DNA and the genotypes by specific pathogen free (SPF)10 polymerase chain reaction (PCR) screening with a general probe hybridisation and INNO-LiPA HPV Genotyping assay, detecting HPV genome in 61% of the cases [2]. Similarly, Guily *et al* (2011) evaluated the type specific HPV prevalence in tonsil histological samples from European patients. The method employed by these authors was the INNO-LiPA Genotyping Extra test, showing a high prevalence of HPV positivity (57%) [13].

High-risk HPV types 16 and 18 are by far the most predominant types at all the anatomical locations reported; however, epidemiological data reveal that the role of HPVs in the etiology of head and neck cancers is rather controversial. The reported frequency of HPV DNA in laryngeal site studies often varies between 3% and 85% in the literature. Works performed by Venuti *et al* (2000) have reported HPV-6 DNA positive cases although in low copy numbers [19], Veitía *et al* (2009) also reported HPV-6 infection in patients with HNSCC by PCR [14]. Major *et al* (2005) showed that based on the prevalence, physical state, genotypes, and copy number of HPV DNA, cancers and papilloma tend to show a different HPV DNA profile [20]. Our results are comparable with those in the literature, since we found that in the infection with a unique genotype, HPV-16 was the most frequent with 18.70%, just as the mixed infection with genotypes HPV 6, 11,16 (18.70%), was followed by genotype HPV-6 (low risk) in 12%, HPV-31 and 51 (high risk) with 4.10% respectively (Table 1).

In relation to the anatomical location, the oral cavity is the localisation with greater HPV positivity (35.40%) followed by the larynx with 29.10%; the genotypes 6, 16, and 51 being the most common in both locations; we also found that 22.5% of the oropharynx cases are high-risk HPV-16 positive, type predominant, and a 8.30% HPV positivity in the paranasal sinus. These data allow us to suggest that HPV could be an important risk factor in oral, laryngeal, and oropharyngeal cancer.

HPV infections as a biological factor have been proposed to play a role in oral carcinogenesis based on epidemiological and clinicalpathological evidence [21]. In the literature, HPV-16 has been identified in 20–90% of the oral carcinomas [22–24]. Guily *et al* showed in their study that the viral infection was present in 10.5% of 209 patients evaluated, being the viral infection more common among females than males [13]. On the other hand, in a multicentre study made by Castillo *et al* (2011) 56% of HPV positivity was detected among 71 samples of oral cavity cancer with the HPV-16 genotype as the most prevalent. Interestingly, the low-risk HPV types 6 and 11 can also be identified in some oral carcinomas, similar to laryngeal carcinomas [25]. This variability in the genotypes rates can be partly explained by variations in the type of sample and different HPV detection methods [26]. Usually, in the majority of the reports high-risk HPV-16 was undoubtedly the predominant viral type detected, and some researchers propose that the presence of HPV-16 in oral exfoliated cells increased the odds of oropharyngeal cancer more than 14-fold [27].

When we evaluated other anatomical locations such as pharyngeal tumours, we observed that these were more frequent among men, and were associated with tobacco and alcohol consumption; however, consistent epidemiological evidences have been extensively obtained over the last decade concerning HPV-associated pharyngeal cancer [28]. The French National Hospital database reports an overall HPV prevalence ranging from 33% to 72% among oropharyngeal cancers [13]. Worldwide, HPV prevalence has been shown to vary from 14–57% in cancers of the oropharynx, however, a lower prevalence was reported in two large case-control studies conducted in central Europe and Latin America in which HPV prevalence was 4.4% among oropharynx cancers, and 3.8% among hypopharynx /larynx cancers [21]. On the other hand, in work carried out by Syrjänen K (2003), they found that of the 322 paranasal sinus carcinomas analysed so far, 70 (21.7%) were positive for some HPV type; these data are comparable with our results [29].

Although the relationship of HPV with squamous carcinoma in the larynx is not well established, it is known that the laryngeal epithelium is susceptible to HPV infection, and the presence of HPV was confirmed by IHC staining to demonstrate the expression of HPV structural proteins [30]. In a meta-analysis performed in 2005, the authors report that laryngeal cancers is the most common HNSCC. HPV DNA was identified in one quarter of tested laryngeal tumours whereas in the normal laryngeal mucosa the reported incidence of HPV infection has been as high as 19%. These suggest that the number of HPV-positive cancers observed might reflect the prevalence of latent HPV infections in the vocal cord epithelium [31]. The HPV prevalence varies from 5% to 41.5% among larynx cancers in different public and private hospitals in France [27], in addition, a systematic analysis of 35 studies from 18 different countries involving a total of 1435 cases of larynx cancer revealed an overall HPV prevalence of 24% [21].

As we said before, in Venezuela there are very few studies that include the features considered in this work, one investigation was made by Liuzzi *et al* (2007), which included the evaluation of 29 samples with HNSCC. HPV infection was detected by PCR in 34.48%, of these the majority were low-risk HPV infection [32]. On the other hand, Mijares *et al* (2007) found the HPV genotype 16 in 24.13% of the samples from oral cancer evaluated during their work [33]; while Jimenez *et al* (1998) showed that in premalignant lesions from the oral cavity, the HPV genotypes predominant were 6, 11, 31, 16, and 32, and they said that these are mostly male and presented in the second and fourth decade of life [34]. These differences in the genotypes found can be explained by the method employed in the HPV detection, and the sample employed in each study (Table 4).

## Conclusion

It is important to highlight that in our study the cases in the more advanced stages presented greater positivity to HPV (57.14%) compared with those that were in the early stages (4.08%). Although our results do not have statistical significance (p>0.05), the evidence suggests that HPV is an important risk factor in HNSCC carcinogenesis as well as in the progression of the tumours.

## Figures and Tables

**Table 1.** HPV genotypes in HNSCC.

Genotype	% HPV positive
16	18.70%
6, 11, 16	18.70%
6	12.00%
6, 51	10.40%
6, 11	4.10%
6, 31	4.10%
31	4.10%
35	4.10%
51	4.10%
11, 16	2.00%
53	2.00%
11, 16, 51	2.00%
16, 18	2.00%
16, 18, 31	2.00%
35, 51	2.00%
6, 16	2.00%
6, 11, 16, 18	2.00%
6, 11, 16, 53	2.00%
6, 31, 33	2.00%

**Table 2.** HPV infection by clinical features.

Clinical features	HPV + INNO-LiPA	Predominant Genotype	Genotype Positivity (%)
**Anatomical Location**			
Oral cavity	35.40%	16	11.76%
		6.51	11.76%
Nostril	0.0%	–	–
Larynx	29.10%	16	14.28%
		6.51	14.28%
Oropharynx	25%	16	22.50%
Sinus paranasal	8.30%	6	50%

**Table 3.** HPV-positive cases according to the size and stage of the tumour.

Tumour Size	Cases	HPV positive
**T1**	(6/71) 8.45%	4.08%
**T2**	(13/71)18.3%	22.44%
**T3**	(21/71) 29.57%	30.61%
**T4**	(31/71) 43.66%	42.85%
**Tumour stage**		
**I**	(5/71) 7.04%	4.08%
**II**	(8/71) 11.2%	14.28%
**II**	(18/71) 25.35%	24.48%
IV^*^	(40/71) 56.33%	57.14%

* Includes the stages IVa and IVb

**Table 4.** Some references.

Author	Method	Results	Reference
Liuzzi *et al* 2007	PCR	100% low risk	*Rev venez Oncol*
Mijares *et al* 2007	PCR	24.13% genotype 16	*Rev venez Oncol*
Jimenez *et al* 2007	PCR	Low risk HPV in benign	*Thesis UCV*
Koskien *et al* 2003	INNO-LiPA	61% HPV positive in HNSCC	*Int J Can*
Guily *et al* 2003	INNO-LiPA	57% HPV positive IN HNSCC	*Head Neck Oncol*
Correnti *et al*	PCR	HPV 16 in Oral Carcinoma	*Clin Cancer Res*
Veitia *et al* 2009	PCR	40% HPV positivity and presence HPV 6 in HNSCC	*Rev venez Oncol*
